# Integrative taxonomy of a new *Odontidium* (Tabellariaceae) species from Poland: Combined morphological and molecular evidence

**DOI:** 10.3897/phytokeys.280.211531

**Published:** 2026-10-14

**Authors:** Julia Sulkowska, Rafał M. Olszyński, Carlos E. Wetzel, Joanna Żelazna-Wieczorek

**Affiliations:** 1 Department of Algology and Mycology, University of Lodz, Łódź, Poland Department of Algology and Mycology, University of Lodz Łódź Poland; 2 Doctoral School of Exact and Natural Sciences, University of Lodz, Łódź, Poland Doctoral School of Exact and Natural Sciences, University of Lodz Łódź Poland; 3 Environmental Sensing and Modelling unit, Biodiversity Monitoring and Assessment group, Luxembourg Institute of Science and Technology – LIST, Belvaux, Luxembourg Environmental Sensing and Modelling unit, Biodiversity Monitoring and Assessment group, Luxembourg Institute of Science and Technology – LIST Belvaux Luxembourg

**Keywords:** Araphid diatoms, Bieszczady National Park, diatom, *
Meridion
*, phylogeny, Polish Carpathians, *rbc*L, springs

## Abstract

Mountain springs are important ecosystems for freshwater biodiversity and may serve as refugia for previously unrecognised diatom diversity. During investigations of springs in the Bieszczady National Park (Poland), a morphologically distinctive taxon of the genus *Odontidium* was observed, which is described here as *Odontidium fontinale* Sulkowska, Żelazna-Wieczorek, Olszyński & Wetzel, **sp. nov**. A morphological and morphometric description was prepared based on analyses conducted using light and scanning electron microscopy. The new species was compared with morphologically similar taxa, including *Odontidium mesodon*, *O. minutum*, *O. rostratum*, and *Tetracyclus rupestre*. *Odontidium fontinale***sp. nov**. is distinguished primarily by its small valve dimensions and a relatively low density of transapical ribs. Furthermore, despite its frequent occurrence in mountain freshwater habitats, the genus *Odontidium* remains poorly known at the molecular level. To address this, molecular data based on *rbc*L sequences from cultures of the new species were obtained to assess its phylogenetic position.

## Introduction

The genus *Odontidium*Kützing 1844 was described based on morphological characters, including the presence of prominent transapical ribs and the mode of colony formation. In early taxonomic interpretations of the genus, *Odontidium* was considered closely related to the previously described *Diatoma*Bory de Saint-Vincent 1824. Initially, the two genera were primarily distinguished based on differences in colony formation and the way cells are connected to one another. In the original description of *Diatoma*, colonies were characterised as being arranged in a zigzag pattern, whereas Kützing’s description of *Odontidium* referred to the formation of short filaments (Bory de Saint-Vincent 1824; Kützing 1844). Williams (1985) proposed additional morphological characters for distinguishing *Diatoma*, such as a raised central sternum and the presence of a ligula on the second girdle band that attaches to the polar position of the valve. At that time, *Odontidium* was treated as a subgenus, and the diagnostic characters included a wide, diffuse sternum, interlocking but non-functional spines, and the structure and number of girdle bands (Williams 1985). However, the taxonomic boundaries between *Odontidium* and *Diatoma* have not been clear and have changed over time (Williams 1985, 1990; Jüttner et al. 2015). Several taxa originally described or classified within *Diatoma* have subsequently been transferred to *Odontidium*. For example, *Diatoma rupestris*, described by Liu et al. (2010), was later transferred to *Odontidium* as *O. rupestre* by Jüttner et al. (2017). Another notable example is *Diatoma mesodon*, a species described by Kützing (1844) in the same work in which *Odontidium mesodon* was described based on Ehrenberg’s material. Williams (1985) detailed the history and difficulties associated with the typification and description of these two species, while also emphasising that colony formation cannot be considered a diagnostic character. *Diatoma mesodon* is currently considered a homotypic synonym of *Odontidium mesodon* (Lange-Bertalot et al. 2017).

The description of new taxa from freshwater habitats has contributed to a better understanding of morphological diversity within the genus *Odontidium* (Levkov and Williams 2011; Jüttner et al. 2017). Nevertheless, species delimitation remains challenging due to the morphological similarity among some taxa and the limited availability of molecular data. At present, publicly available molecular data for *Odontidium* remain extremely limited. In particular, molecular data for the *rbc*L marker, which is widely used in diatom phylogenetic studies for species resolution, are lacking for this genus, in contrast to the related genus *Diatoma* (Theriot et al. 2010; Fray et al. 2024). This lack of molecular data has so far limited the possibility of evaluating species boundaries and phylogenetic relationships within *Odontidium* using molecular evidence.

Species of the genus *Odontidium* are frequently reported from cold, oligotrophic streams and spring habitats, particularly in alpine and other high-elevation regions (Cantonati et al. 2012, 2022; Jüttner et al. 2017). Despite its relatively frequent occurrence in these environments, the taxonomy and species diversity of the genus remain insufficiently resolved. Spring ecosystems represent particularly important habitats for investigating diatom diversity and taxonomy. Numerous diatom species have been described from springs (Żelazna-Wieczorek 2011; Cantonati et al. 2016; Beauger et al. 2017, 2022; Lai et al. 2018, 2025), further highlighting their importance as habitats with substantial and still incompletely documented diatom diversity.

During investigations of spring habitats within the Bieszczady Mountains, populations of *Odontidium* exhibited a combination of morphological features not corresponding to any species described to date. To evaluate their taxonomic status, detailed morphological observations and molecular analyses were conducted using two independent strains of the potentially new taxon, three strains of *Odontidium mesodon*, and representatives of the genus *Meridion* Agardh.

The aims of the present study were to describe the new species *Odontidium fontinale* in comparison with closely related and morphologically similar taxa, to determine its phylogenetic position based on the first molecular data for the genus *Odontidium* using maximum likelihood (ML) analysis, and to discuss the implications of the obtained results for taxonomy and phylogenetic relationships.

## Materials and methods

### Field research

The Bieszczady National Park, established in 1973, is the largest national park in the Polish Carpathians and protects some of the best-preserved mountain ecosystems in Central Europe. Together with adjacent protected areas in Slovakia and Ukraine, it forms part of the East Carpathian Biosphere Reserve, a UNESCO transboundary reserve recognised for its exceptional natural value (Winnicki and Zemanek 2001).

Springs are prevalent landscape elements of the Bieszczady Mountains and support diverse diatom assemblages, including rare, threatened, and habitat-specialised taxa (Żelazna-Wieczorek 2012; Żelazna-Wieczorek and Knysak 2017; Żelazna-Wieczorek and Mostowik 2019; Żelazna-Wieczorek and Sulkowska 2024).

**Figure 1.**
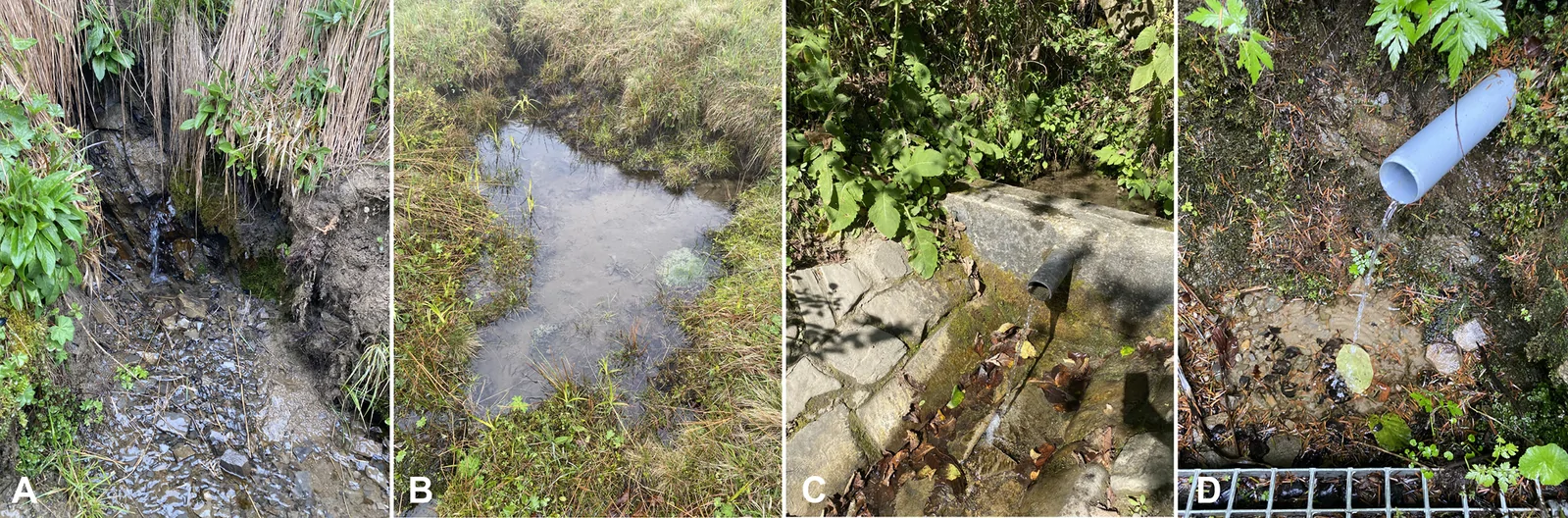
Locations of the studied springs in the Western Bieszczady Mountains. **A**. Spring at Goprowska Pass; **B**. Spring on the southern slope of Połonina Wetlińska; **C**. Dwerniczek Spring; **D**. Tarnawa Spring.

The study area is located in south-eastern Poland within the Western Bieszczady Mountains, a part of the Eastern Carpathians. Algological material was collected in May and September of 2024 and 2025 from four spring habitats, three situated within the Bieszczady National Park and one located in its buffer zone (Fig. 1; Table 1).

**Table 1.** Characteristics of the study sites. Values are presented as ranges recorded during the period 2019–2025. An asterisk (*) indicates values measured at the time of collection of material used to establish cultures.

**Spring**	**Elevation [m a.s.l.]**	**Located within National Park**	**Spring type**	**Hydromorphological modification**	**Water temperature [°C]**	**pH**	**Electrolytic conductivity [µS cm^-1^]**	**Coordinates**
Spring at the Goprowska Pass	1174	Yes	Rheocrenic	Natural	5.4–14.3	7.60–8.55	116–177	49.08084°N, 22.72991°E
					5.5*	7.9*	160*	
Spring at the Połonina Wetlińska – south slope	1149	Yes	Limnocrenic	Natural	5.6–11.7	6.00–8.10	91–178	49.15931°N, 22.54528°E
					6.1*	6.0*	91*	
Tarnawa Spring	722	Yes	Rheocrenic	Modified	6.8–10.0	7.05–7.84	247–350	49.12368°N, 22.78946°E
					9.4*	7.8*	350*	
Dwerniczek Spring	604	Buffer zone	Limnocrenic	Modified	8.2–9.8	7.20–7.95	210–318	49.21225°N, 22.67230°E
					9.6*	7.2*	253*	

At each spring, algological material was collected from two substrates: sandy sediments and submerged mosses. Both substrate types were sampled separately at each of the four springs. Epipsammic samples were collected from the uppermost sediment layer using a glass pipette, whereas samples from mosses were collected with a disposable toothbrush. Samples from the two microhabitats were collected separately, placed in separate sterile 125 mL containers, and transported in a refrigerator at approximately 9 °C to the Department of Algology and Mycology, University of Lodz, for further processing. The environmental samples were divided into two parts, one for cultivation and one for preparation. The water temperature, pH, and electrolytic conductivity were measured *in situ* during field sampling using CC-411 and CP-401 devices (ELMETRON, Poland). The recorded values are summarised in Table 1. Field sampling within the Bieszczady National Park was conducted under appropriate permits issued by the Park Authority (Nos 2/24 and 21/25).

### Culturing methods

Samples were transported to the laboratory of the Department of Algology and Mycology, University of Lodz, for further processing and analysis. Single diatom cells were isolated from the samples using a glass micropipette under a Nikon Eclipse TS100 E Plan APO 10×/0.25 WD 7.0 inverted light microscope (Nikon, Japan). Each isolated cell was transferred to a sterile plastic Petri dish (6 cm diameter) containing sterile Bacillariophycean Medium (Diat. Medium ver. 10.2008; SAG Culture Collection of Algae, University of Göttingen) and cultivated at 6 °C under a 12 h light: 12 h dark photoperiod to obtain monocultures. After successful establishment, monoclonal cultures were transferred to 25 cm^²^ culture flasks containing fresh medium and grown under the same conditions until sufficient biomass was available for morphological examination and DNA extraction. Using this procedure, cultures were established for five *Odontidium* strains and two *Meridion circulare* strains (Suppl. material 1).

### Microscopy analysis

To obtain cleaned diatom frustules, the environmental samples were treated according to the procedure outlined by Żelazna-Wieczorek (2011). The cultured material was oxidised using the same reagents, but the time for which the material was kept in the acids was reduced to one day due to the low organic matter content of the material. In total, eight microscope slides were prepared from the environmental material, representing the four investigated springs. Two slides were prepared from each spring, one from sandy sediment and one from submerged mosses. Seven additional microscope slides were prepared from the cultured material, with one slide prepared from each strain. Additionally, the type material of *Odontidium minutum* Levkov & Williams, 2011 from North Macedonia was included in the analyses, and one microscope slide was prepared from this material (accession no. MKNDC000407). The purified material was applied to coverslips and, after drying, mounted onto glass microscope slides in Naphrax resin (Brunel Microscopes Ltd., UK). The taxonomic and morphometric analyses were performed with a Nikon Eclipse E600 microscope at 1000× magnification (plan oil–immersion objective 100×/1.40 Plan APO DIC H). In total, 16 permanent slides were analysed, including eight prepared from environmental material, seven from cultured strains, and one from the type material. The morphological analysis of *Odontidium* taxa was performed based on environmental material and cultured material from strains. Morphometric measurements of valves (*n* = 295, Suppl. material 2) were carried out on environmental, cultured, and type material to assess the morphological variability of the studied taxa. The following features were taken into account: valve length, width, and the number of transapical ribs in 10 µm. Based on Euclidean distance, non-metric multidimensional scaling (nMDS) analysis was performed. To analyse differences in valve measurements, the one-way ANOSIM test for the Species and Environment factors was used. PRIMER 7 software (PRIMER-E Ltd, UK) was used for the analysis.

An OPTA-TECH digital camera (resolution 3840 × 2040, 1/2.7" SONY sensor) was used to obtain light microscopy (LM) images of cleaned frustules from cultured and environmental samples. For scanning electron microscopy (SEM), parts of the oxidised suspensions were filtered with additional deionised water through a 3 µm Isopore™ polycarbonate membrane filter (Merck Millipore), pieces of which were affixed to aluminium stubs after air-drying. Filters were coated with a 12 nm platinum layer using a Leica EM ACE600 sputter coater (Leica Microsystems GmbH, Germany). SEM analysis was performed with an ultra-high-resolution analytical field-emission scanning electron microscope (FE-SEM; Hitachi SU-70, Hitachi High-Technologies Corporation, Tokyo, Japan) at 5 kV and a 10 mm working distance, using the lower secondary electron (SE-L) detector.

### DNA isolation, amplification, and sequencing

Total DNA isolation from diatom monoclonal cultures was performed using the Chelex 100 (Bio-Rad, CA, USA) method described by Dąbek et al. (2017). The diatom biomass was obtained from 1.5 mL of cell suspension after centrifugation for 5 min at 5,000 rpm. The pellet was incubated with 200 µL of 10% Chelex 100 working solution at 95 °C for 20 min with continuous shaking. Next, the samples were centrifuged for 5 min at 5,000 rpm to remove cell debris and Chelex 100 resin. The DNA-containing supernatant was transferred to a new sterile 1.5 mL Eppendorf tube and stored at –20 °C for further analysis. The plastid *rbc*L gene was amplified by PCR using the primers and amplification protocol described by Olszyński et al. (2024). PCR amplifications were performed in several replicates to obtain high-quality products, which in turn were visualised on a 1% agarose gel (Sigma-Aldrich, Germany) and stained with ethidium bromide (Sigma-Aldrich, Germany). PCR products were purified and sequenced by SEQme s.r.o. (Czech Republic) using Sanger sequencing with the primers used for amplification. The obtained sequences were assembled in Geneious Prime 2026.0.2 (Biomatters Inc., MA, USA). The newly generated sequences were deposited in the NCBI GenBank database (https://www.ncbi.nlm.nih.gov).

### Phylogenetic analyses

Phylogenetic analysis was performed using the *rbc*L marker. The dataset comprised 83 sequences, and the final alignment consisted of 955 bp. *Eunotia bilunaris* (Ehrenberg) Schaarschmidt was used as the outgroup. Sequences were aligned using MAFFT v. 7 and manually inspected, with minor corrections introduced where necessary (Suppl. material 3). The best-fitting evolutionary model was selected according to the Akaike information criterion (AIC) using MEGA 11. ML analysis was performed in IQ-TREE version 3 using the same settings as described by Olszyński et al. (2026), including the ultrafast bootstrap (UFBoot) algorithm with 10,000 replicates and 10,000 bootstrap alignments. Voucher information and GenBank accession numbers for all sequences included in the analysis are provided in Suppl. material 4.

## Results

### Taxonomy

**Class: Fragilariophyceae Round, 1990**

**Order: Tabellariales Round, 1990**

**Family: Tabellariaceae Kützing, 1844**

**Genus: *Odontidium* Kützing, 1844**

#### Odontidium fontinale

Taxon classificationPlantaeLittorinimorphaCaecidae

Sulkowska, Żelazna-Wieczorek, Olszyński & Wetzel sp. nov.

CDE89152-BE27-5AA7-B78D-D05D09335E6F

Figs 2A–Z ’’, 3–5

##### Morphological description.

**Figure 2.**
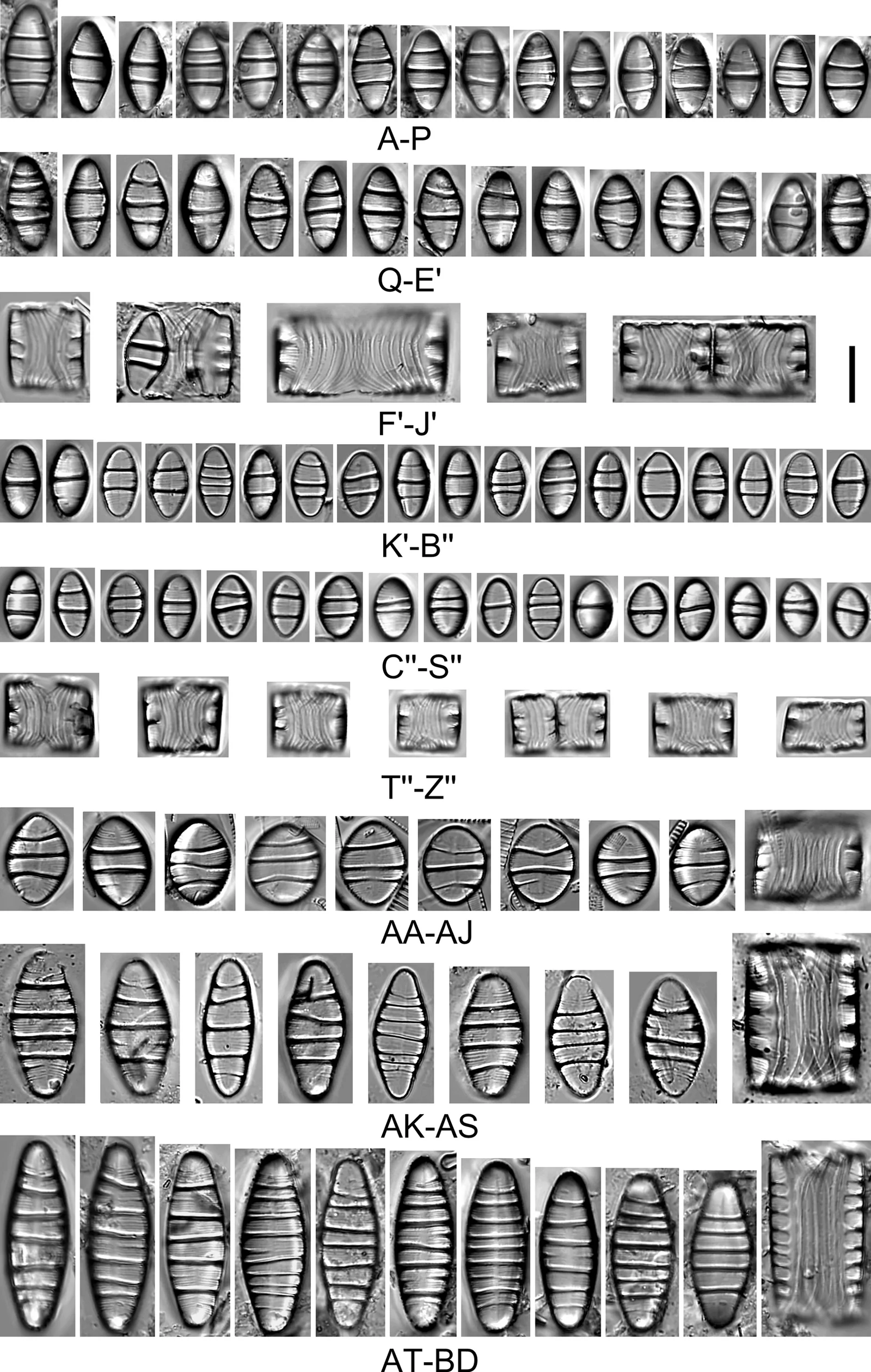
Light microscope photomicrographs of *Odontidium fontinale* sp. nov. and *Odontidium* spp. **A–J'**. *O. fontinale* sp. nov. from a natural population; **A–E'**. Valve view; **G**. Holotype; **F'–J'**. Girdle view. **K'–Z"**. *O. fontinale* sp. nov. from culture; **K'–S'**. Valve view; **T'–Z"**. Girdle view. **AA–AJ**. *Odontidium minutum* from type material; **AA–AI**. Valve view; **AJ**. Girdle view. **AK–BD**. *Odontidium mesodon* from a natural population; **AK–AS**. Specimens from Dwerniczek Spring; **AK–AR**. Valve view; **AS**. Girdle view; **AT–BD**. Specimens from the spring at Goprowska Pass; **AT–BC**. Valve view; **BD**. Girdle view. Scale bar: 10 μm.

**Figure 3.**
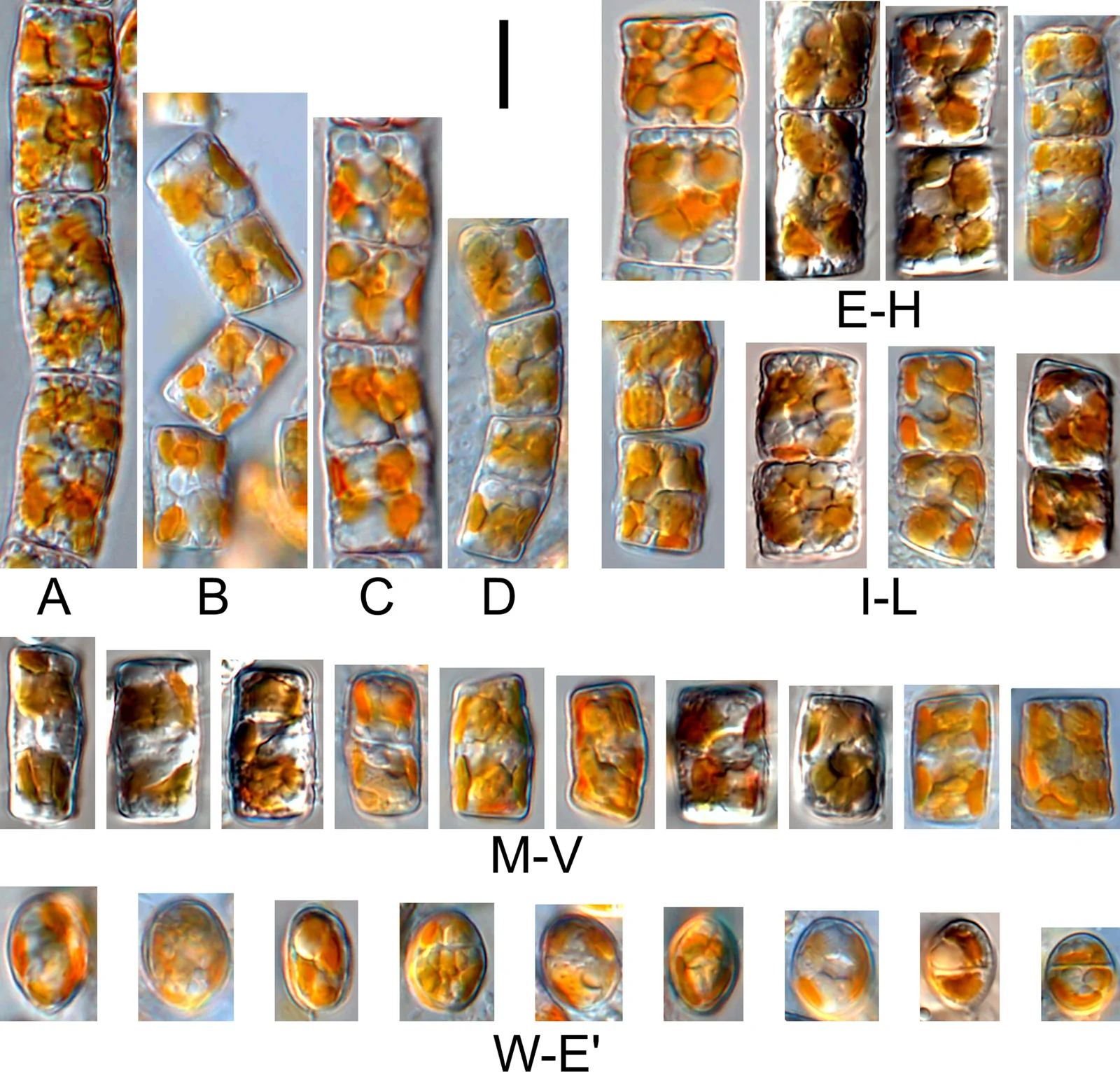
DIC light microscope photomicrographs of *Odontidium fontinale* sp. nov. from culture. **A–V**. Girdle view; **A–L**. Chains of multiple cells; **M–V**. Single cells; **W–E'**. Valve view. Scale bar: 10 μm.

**Figure 4.**
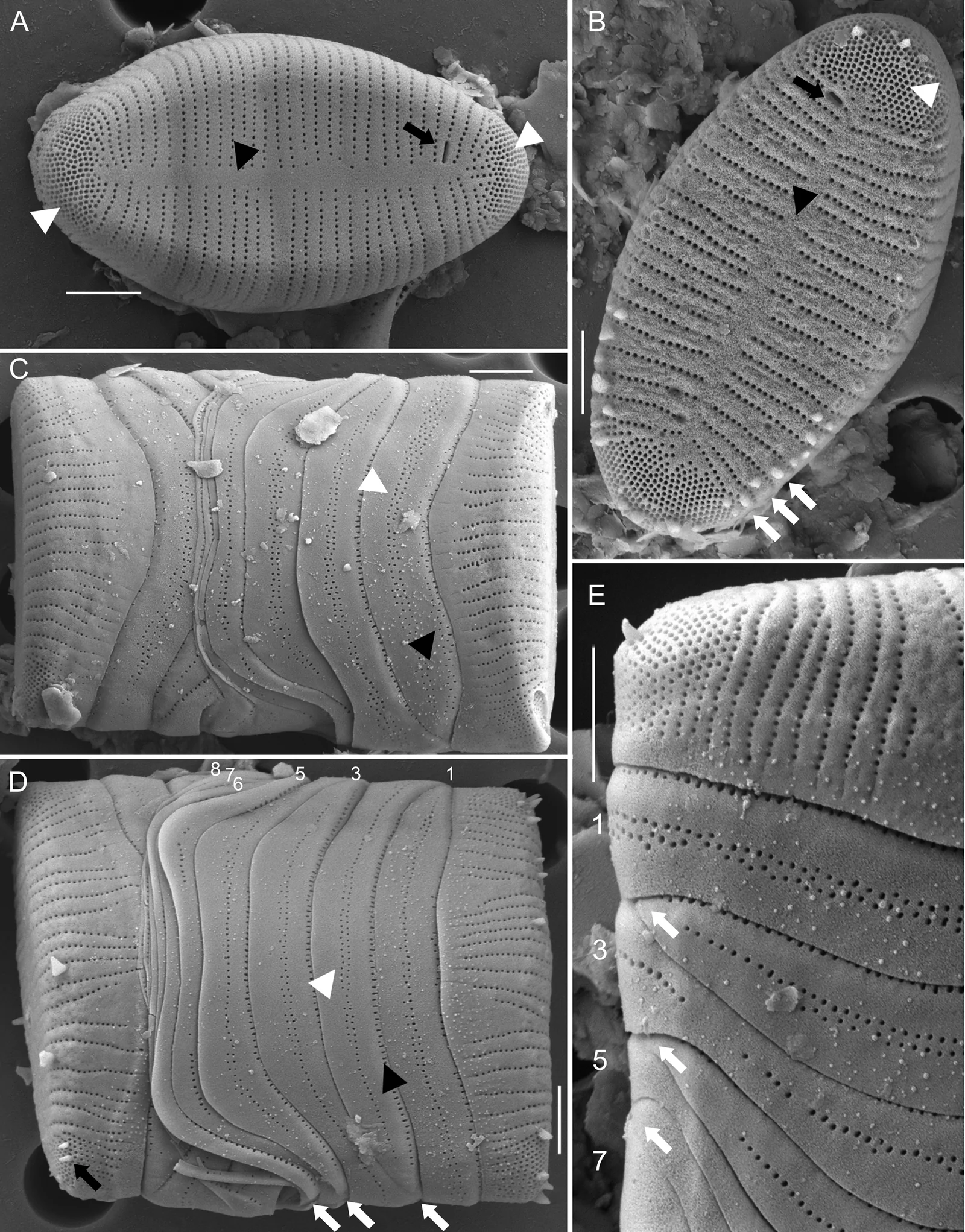
SEM photomicrographs of *Odontidium fontinale* sp. nov., external view. **A, B**. Valve face showing a narrow sternum (black arrowhead). At one apex, adjacent to the sternum and apical pore field (white arrowhead), a rimoportula with an elongate slit-like opening is visible; **C–E**. Girdle view; **C, D**. Striae extending from the valve face onto the mantle. Advalvar poroids, located at the junction between the *pars interior* and *pars exterior* (black arrowheads), usually form a single row of elongate pores (**D**), but occasionally two rows are present (**C**). Abvalvar poroids consist of one or two rows of rounded pores (white arrowheads); **D**. Cingulum composed of numerous copulae; alternating copulae are open on one side (white arrows) and closed on the opposite side; **E**. Detail of the cingulum, showing the closed ends of bands 1, 3, 5, and 7 (arrows) and the open ends of bands 2, 4, and 6.

**Figure 5.**
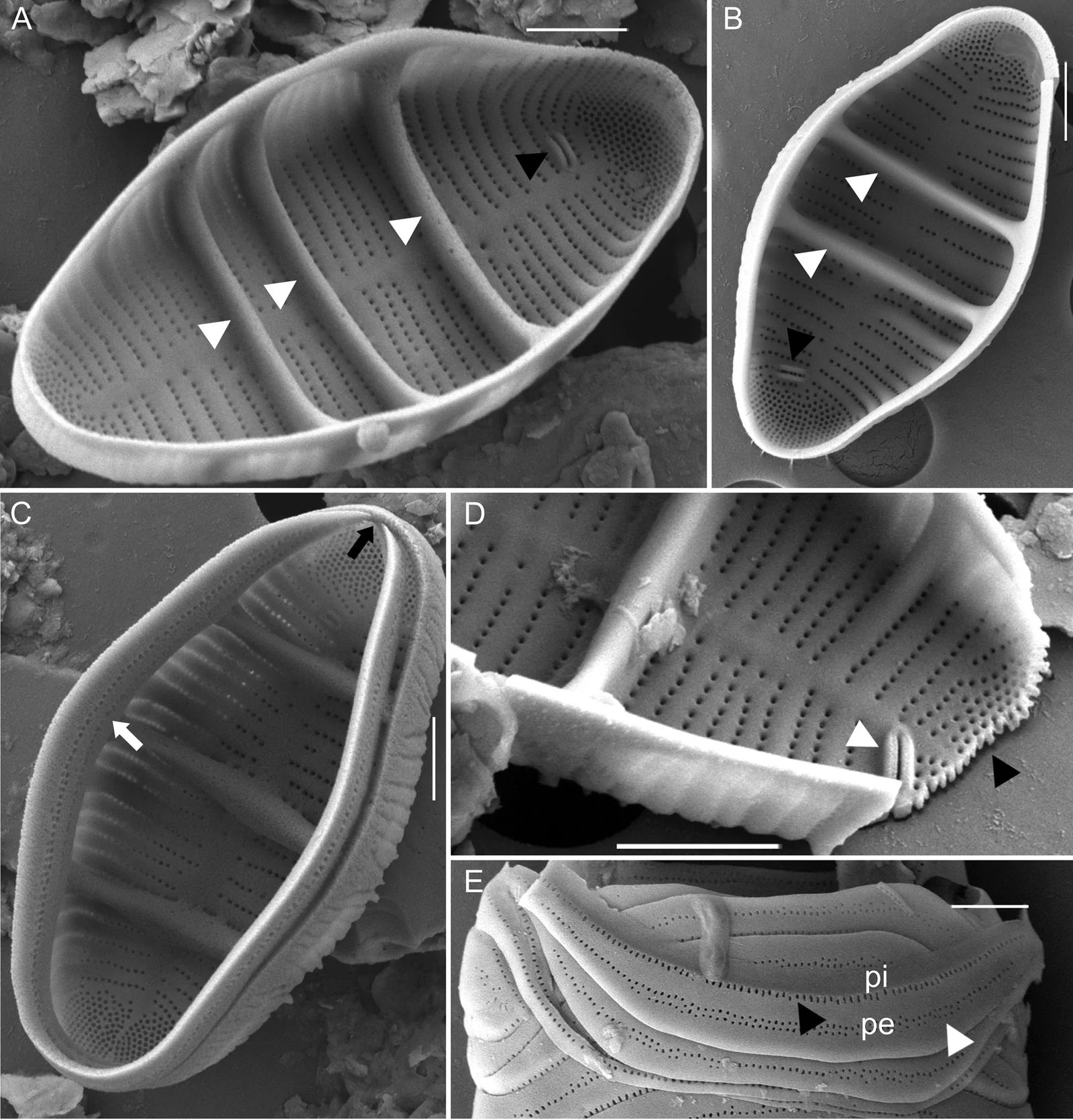
SEM photomicrographs of *Odontidium fontinale* sp. nov., internal and external views. **A–C**. Internal valve views showing prominent transapical ribs (white arrowheads); **A, B**. A labiate process is present near one apex (black arrowheads); **C**. Oblique internal view showing the valvocopula, with internally visible poroids (white arrow) and an open end (black arrow); **D**. Valve apex showing the internally projecting labiate process (white arrowhead) and a cross-section of the valve face, showing the areolae as perforations extending through the siliceous valve wall (black arrowhead); **E**. External view of the cingulum, showing the *pars interior* (pi), *pars exterior* (pe) of the copulae, advalvar poroids (black arrowhead), and abvalvar poroids (white arrowhead).

Colonies most commonly form short filaments (Fig. 3A, C–L), occasionally forming a slightly zigzag pattern (Fig. 3B). In LM, four to eight elliptic, lobed plastids were observed within living cells (Fig. 3M–E'). Frustules are rectangular in girdle view (Figs 2F'–J', T"–Z", 3A–V). Valves are isopolar, elliptic to elliptic-rhombic, with rounded apices (Figs 2A–E', K'–S", 3W–E'). Valve dimensions (*n* = 130): length 9–16 µm, width 5–7 µm. Transapical ribs are nearly all primary; secondary ribs are rarely present, 1–3 (4) in 10 µm (*n* = 130). Striae are visible in LM, although often difficult to resolve. In SEM, striae are uniseriate, 20–28 in 10 µm (*n* = 75). Striae between ribs are parallel or at a slight angle towards the poles, with 3–9 rows between ribs (*n* = 75). One rimoportula is present per valve, located near the pore field (Figs 4A, 4B, 5A–D). A simple apical pore field is present at each pole, composed of round porelli. The sternum is narrow and does not extend into the apical pore field (Fig. 4A, B). In some valves, spines are lacking (Fig. 4A, C). When present, spines are located on the virgae (Fig. 4B). Virgae are of varying width; vimines are much shorter than virgae (Fig. 4A, B). The cingulum is composed of two to eight copulae (Fig. 4D). The valvocopula has two or three rows of poroids on the *pars exterior*. Poroids located at the junction between the *pars interior* and *pars exterior* usually form a single row of elongate pores, rarely two rows (Figs 4C, 4D, 5C). Copulae have a row composed of multiple series of poroids on the *pars exterior* (Figs 4C–E, 5C, 5E).

##### Type locality.

Poland, Subcarpathian region, from a circumneutral, weakly to moderately mineralised freshwater spring at the Goprowska Pass (49.08084°N, 22.72991°E), collected on 22 May 2025.

***Holotype***: A permanent slide containing valves of *O. fontinale* sp. nov. is deposited in the University of Lodz Algae Collection under accession ZBdPN_GOPRII22052025. The holotype is illustrated in Fig. 2G (designated here).

***Isotype***: A permanent slide under accession SZCZ 29881 is deposited in the Szczecin Diatomological Collection at the University of Szczecin, Poland.

##### Etymology.

The specific epithet “*fontinale*” refers to the spring habitats (Latin: *fons*, *fontis* – spring) from which the species was collected and where it appears to be most abundant.

##### Comparison of morphological features.

**Figure 6.**
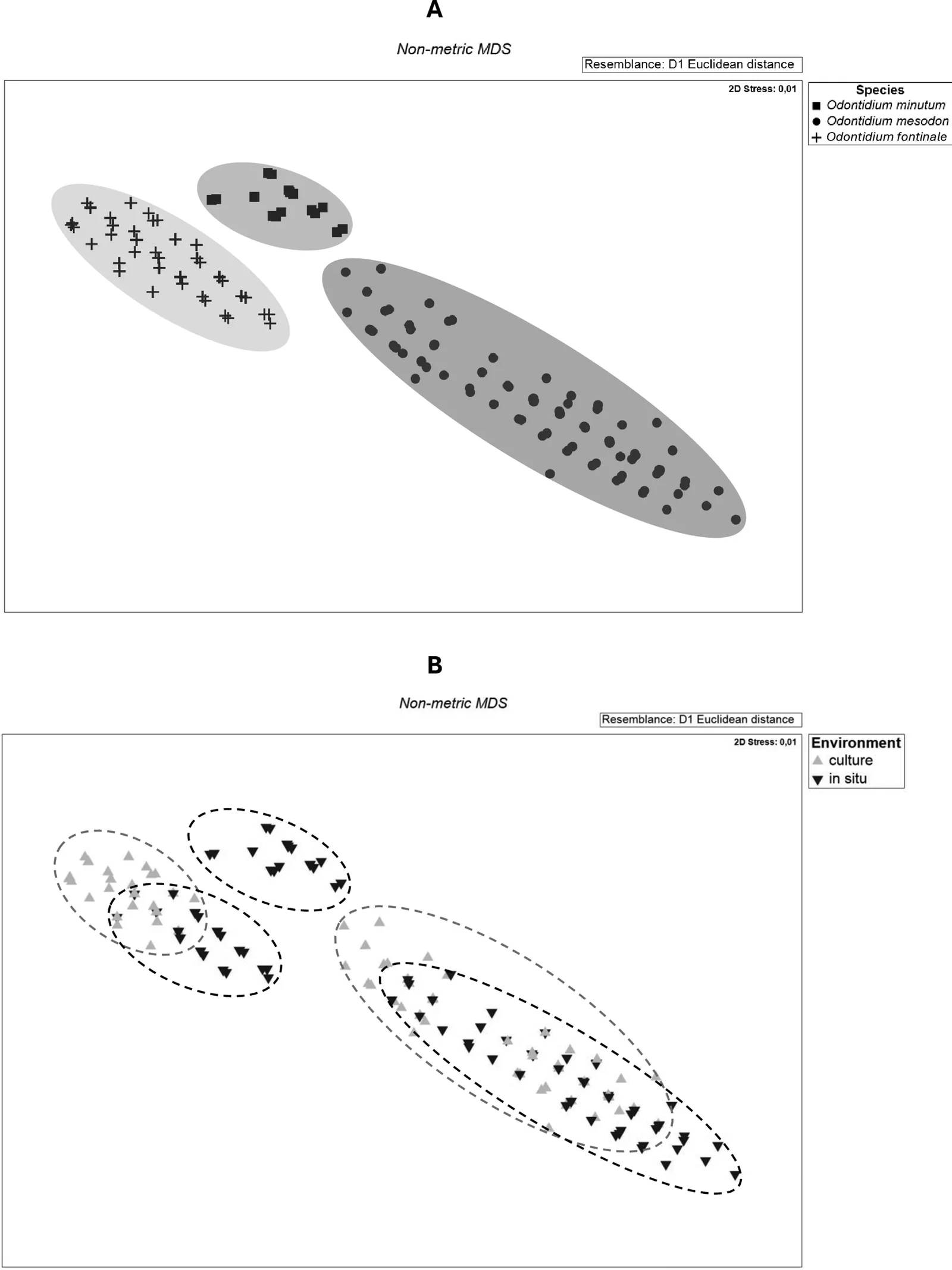
Analysis of valve morphology of three *Odontidium* species based on Euclidean distance. **A**. Morphometric differentiation between taxa; **B**. Morphometric differentiation between environments. Two-dimensional nMDS plot.

For comparative analysis of the main morphological features, nMDS (stress = 0.01) was performed on 295 valves representing *Odontidium* species. The analysis showed three different morphometric groups among the analysed species. First, *O. fontinale* formed a clearly distinct cluster, well separated from *O. mesodon* and *O. minutum* (Fig. 6A). Among the analysed taxa, *O. mesodon* exhibited the greatest morphological variability. An additional nMDS analysis comparing cultured and environmental material demonstrated that valves obtained from monoclonal cultures were generally smaller than those observed in environmental samples. Despite this size reduction, specimens of each species remained clearly separated, indicating that the observed interspecific differences were maintained under culture conditions (Fig. 6B). The ANOSIM test for differences in valve dimensions indicated differences for the Species factor (*R* = 0.859, *p* = 0.001) and the Environment factor (*R* = 0.061, *p* = 0.001). A summary of the morphometric measurements obtained from cultured strains is provided in Suppl. material 5, whereas complete morphometric measurements of all analysed valves used for the nMDS analysis are provided in Suppl. material 2.

##### Taxonomic comments.

**Figure 7.**
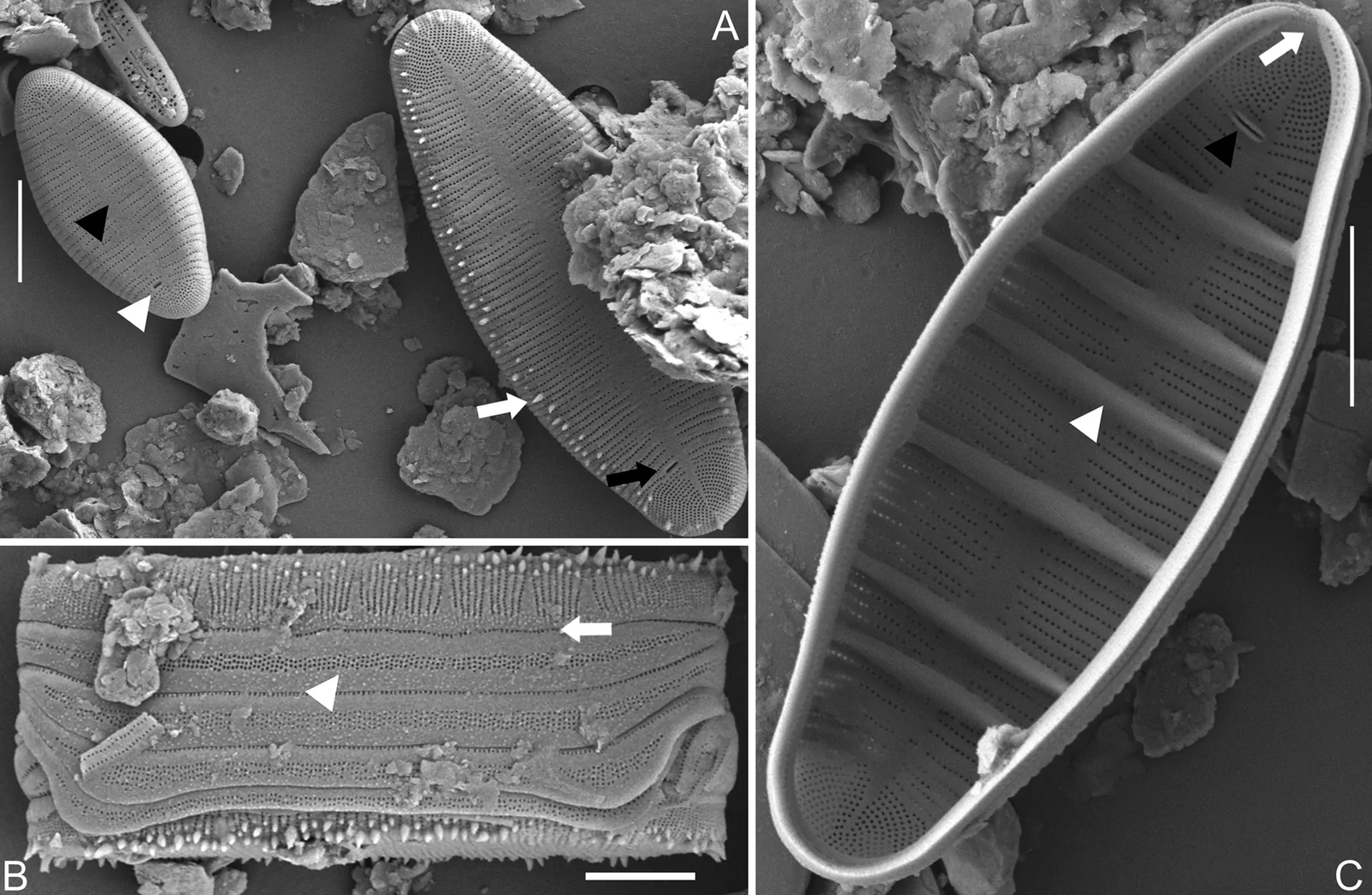
SEM photomicrographs of *Odontidium fontinale* sp. nov. and *O. mesodon*. **A**. External valve views of *O. fontinale* sp. nov. (left), showing the sternum (black arrowhead) and external slit-like opening of the rimoportula (white arrowhead), and *O. mesodon* (right), showing the rimoportula opening (black arrow) and marginal spines (white arrow); **B**. Girdle view of *O. mesodon* showing cingulum ornamentation, including advalvar and abvalvar poroid rows (arrow and arrowhead, respectively); **C**. Internal view of *O. mesodon* showing prominent transapical ribs (white arrowheads), a labiate process (black arrowhead), and the open end of the valvocopula (arrow).

Morphologically, *Odontidium fontinale* most closely resembles *O. mesodon* (Fig. 2AK–BD, Suppl. material 6), a species frequently reported from springs and oligotrophic running waters (Cantonati et al. 2006). Both taxa share a similar valve outline and the presence of prominent transapical ribs. However, *O. fontinale* differs in its generally smaller valve width, 5–7 µm compared with 8–11 µm in *O. mesodon*, and in the lower density of transapical ribs, 1–3 in 10 µm in *O. fontinale* sp. nov. compared with 3–6 in 10 µm in *O. mesodon* (Lange-Bertalot et al. 2017). SEM observations also revealed a difference between these two taxa. The axial area also differs: in *O. fontinale*, the sternum does not extend into the pore field, whereas in *O. mesodon* it reaches almost to the end of the valve (Fig. 7). Another morphologically similar taxon is *O. minutum*, originally described from spring habitats in North Macedonia (Levkov and Williams 2011). In comparison with *O. fontinale* sp. nov., *O. minutum* possesses distinctly broader valves, 9–11 µm in width compared with 5–7 µm (Figs 2AA–AJ, 6). A further morphologically similar species also described from springs is *O. rostratum* Levkov & Jüttner, 2015. Although smaller specimens of this taxon may overlap in size with *O. fontinale*, *O. rostratum* can be distinguished by its distinctly rostrate valve apices (Jüttner et al. 2015). In contrast, the valve ends of *O. fontinale* remain broadly rounded throughout the observed size range.

Due to its small size and strongly silicified transapical ribs, *O. fontinale* may also be confused under light microscopy with *Tetracyclus rupestre* (Kützing) Grunow. However, *T. rupestre* differs in its arrangement of striae, structure of the girdle bands, and presence of septa. In addition, the position and morphology of the rimoportula differ between the two taxa (Williams et al. 2021).

In comparison with morphologically similar taxa, *Odontidium fontinale* is distinguished by its relatively small valve dimensions and a lower density of transapical ribs. In the studied populations, two ribs in 10 µm were most commonly observed, whereas higher rib densities are typically reported for *O. mesodon*, *O. minutum*, and *O. rostratum* (Table 2).

**Table 2.** Comparison of morphological features of *Odontidium fontinale* sp. nov. with morphologically related taxa.

**Taxa**	***O. fontinale* sp. nov**.	***O. mesodon* (Ehrenberg) Kützing**	***O. mesodon* (Ehrenberg) Kützing**	***O. minutum* Levkov & Williams**	***O. rostratum* Levkov & Jüttner**	***T. rupestre* (Kützing) Grunow**
Length [µm]	9–16	10–40	18–33	12–16	10–58	4–28
Width [µm]	5–7	6–14	8–11	9–11	4.5–9	2–3*; 3–12**
Striae [in 10 µm]	20–28	22–25	21–26	24–30	n.d.	18–22
Transapical ribs [in 10 µm]	1–3 (4)	3–6	3–5	2–5	3–4	3–5
Rimoportula	1	1 or 2	1 or 2	1	1	1
Spines	Absent or 1 row	Present	Present	Present	Present	Present
References	This study	Lange-Bertalot et al. 2017	This study	Levkov and Williams 2011	Jüttner et al. 2015	Williams et al. 2021*; Lange-Bertalot et al. 2017**

##### Habitat and ecology.

*Odontidium fontinale* has so far been recorded from two of the four investigated spring ecosystems in the Western Bieszczady Mountains (Polish Carpathians). These two ecosystems, Goprowska Pass and Tarnawa, are geographically isolated from each other. The species was found in springs characterised by relatively low water temperatures, ranging from 5.4–14.3 °C, with pH values ranging from 7.05–7.94 and electrolytic conductivity ranging from 116–350 µS cm^-1^ (Table 1). The two springs represent contrasting light conditions, ranging from high light availability at Goprowska Pass to strong shading at Tarnawa Spring. At both springs, *O. fontinale* occurred on both investigated substrates. However, the species was considerably more abundant on bryophytes than in sandy sediments. The accompanying diatom assemblages differed between the two springs. At Goprowska Pass, *Odontidium fontinale* co-occurred with *Achnanthidium minutissimum* (Kützing) Czarnecki, *Odontidium mesodon* (Ehrenberg) Kützing, *Fallacia insociabilis* (Krasske) Mann, *Humidophila perpusilla* (Grunow) Lowe, Kociolek, Johansen, Van de Vijver, Lange-Bertalot & Kopalová, and *H. contenta* (Grunow) Lowe, Kociolek, Johansen, Van de Vijver, Lange-Bertalot & Kopalová. At Tarnawa Spring, *Odontidium fontinale* co-occurred with *Achnanthidium minutissimum*, *Cocconeis lineata* Ehrenberg, *Halamphora normanii* (Rabenhorst) Levkov, and *Amphipleura pellucida* (Kützing) Kützing.

##### Molecular phylogeny.

**Figure 8.**
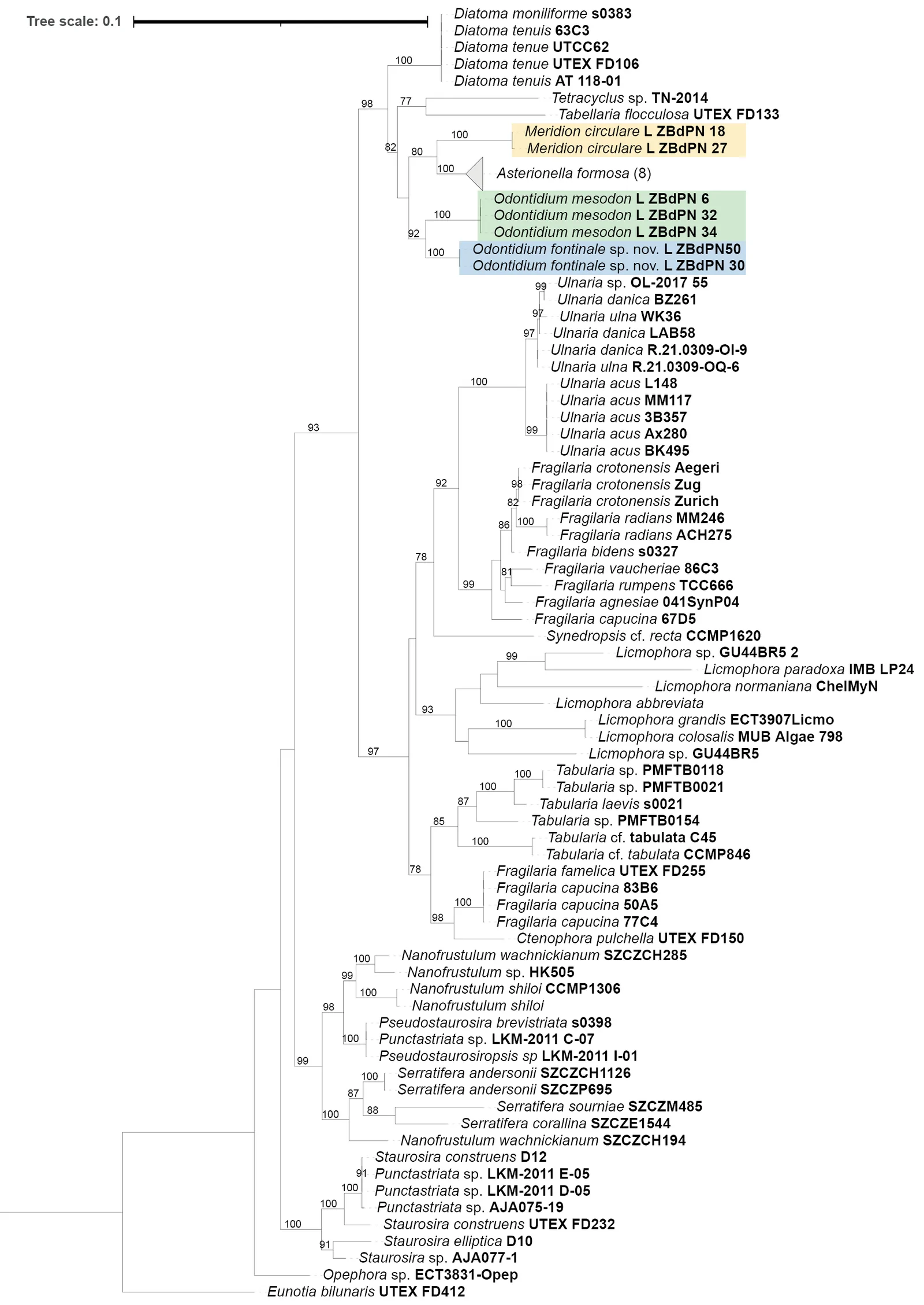
Phylogenetic tree of Fragilariophyceae, with *Eunotia bilunaris* used as the outgroup, based on the chloroplast *rbcL* marker (955 bp) and reconstructed using ML. Sequences obtained in the present study are highlighted by coloured boxes: *Odontidium fontinale* sp. nov. in blue, *O. mesodon* in green, and *Meridion circulare* in yellow. Numbers above the branches indicate ML bootstrap support values; only values ≥75 are shown.

The ML phylogenetic reconstruction based on the *rbc*L marker placed the two strains of *Odontidium fontinale*, L ZBdPN30 and L ZBdPN50, in a distinct and maximally supported clade (Fig. 8, blue box). The three strains identified as *O. mesodon* (L ZBdPN6, L ZBdPN32, and L ZBdPN34) also formed a separate clade with maximum bootstrap support (Fig. 8, green box). The *O. fontinale* clade was recovered as sister to the *O. mesodon* clade, and together these lineages formed a strongly supported monophyletic *Odontidium* group (ML = 92). Thus, although the newly described species was most closely related to *O. mesodon* among the taxa included in the analysis, its strains formed a separate, well-supported evolutionary lineage. These results support both the distinction of *O. fontinale* from *O. mesodon* and its placement within the genus *Odontidium*.

The phylogenetic analysis also included two newly obtained *Meridion circulare* sequences, represented by strains L ZBdPN18 and L ZBdPN27. Both strains clustered together in a maximally supported clade (Fig. 8, yellow box). The *M. circulare* lineage was grouped with the collapsed clade comprising eight strains of *Asterionella formosa*, with moderate bootstrap support (ML = 80). The *Meridion*–*Asterionella* lineage was recovered close to the *Odontidium* clade in the phylogenetic tree, enabling the relationships among these freshwater araphid diatom lineages to be examined within a broader molecular context.

##### Gene sequences.

Sequences (*rbc*L) were deposited in GenBank: L_ZBdPN_6 (PZ727227), L_ZBdPN_18 (PZ727228), L_ZBdPN_27 (PZ727229), L_ZBdPN_30 (PZ727230), L_ZBdPNA_32 (PZ727231), L_ZBdPN_34 (PZ727232), and L_ZBdPN_50 (PZ727226).

## Discussion

The taxonomy of *Odontidium* has undergone substantial revision in recent decades. These revisions involved the re-examination of type material and the assessment of morphological characters used for species identification (Williams 1985; Jüttner et al. 2015). One of the characters proposed as important for the diagnosis of the subgenus *Odontidium* was the presence of spines (Williams 1985, 1990). In the present study, spine development was found to be variable within *Odontidium fontinale*. The occurrence of valves both with and without spines indicates that the presence of marginal spines is variable and should not be treated as a diagnostic feature. Interestingly, valves without spines predominated in samples from surface sediment from the Goprowska Pass Spring. Specimens with well-developed spines occurred predominantly on bryophytes. This observation supports previous suggestions that spine development may be more variable than previously assumed. Jüttner et al. (2017) documented *Odontidium* species lacking spines from the Himalaya. The results further emphasise the need to evaluate morphological characters within a broader phylogenetic framework.

The identification and description of diatom species have been based primarily on morphological features observed using light and electron microscopy. Although detailed morphological analyses remain fundamental to diatom taxonomy, the increasing availability of molecular data has substantially expanded the possibilities for species delimitation and the reconstruction of phylogenetic relationships (Kahlert et al. 2019). Integrative approaches combining morphological and molecular evidence are therefore increasingly applied in diatom taxonomy, particularly when morphologically similar taxa are difficult to distinguish based only on morphology (Li et al. 2022; Olszyński et al. 2024; Rybak et al. 2026).

The molecular data presented here provide the first phylogenetic insight into the genus *Odontidium* and contribute to a better understanding of relationships within the Tabellariaphycidae. The present analyses provide molecular evidence based on *rbc*L for *Odontidium* and should be regarded as an important first step towards resolving relationships within the genus. The significance of molecular data for araphid diatom taxonomy has been emphasised in several recent studies (Sato 2008; Li et al. 2016). Medlin and Desdevises (2016) demonstrated that molecular phylogenetic analyses provide an important framework for understanding evolutionary relationships within this morphologically diverse group. Interestingly, the genus *Odontidium* was not included among the taxa assigned to the clade containing *Asterionella*, *Diatoma*, and *Tabellaria*, reflecting the scarcity of available molecular data for the genus at that time (Medlin and Desdevises 2016). Similarly, the recent higher-level classification proposed by Kociolek et al. (2026) placed *Odontidium* within the Tabellariaphycidae largely on the basis of morphological evidence, further illustrating the need for additional sequence data from representatives of the genus and related taxa. The molecular data presented in this study contribute not only to species delimitation between *Odontidium mesodon* and *O. fontinale* sp. nov. but also to improving the understanding of phylogenetic relationships within the family Tabellariaceae. *Odontidium* has traditionally been regarded as the genus most closely related to *Diatoma*. However, the molecular phylogeny indicates a closer relationship between *Odontidium* and the genera *Meridion* and *Asterionella* than with *Diatoma*. Although the primary focus of the present study was the description of *O. fontinale*, the incorporation of the *Meridion* strains expands the available molecular reference data for the genus and contributes to a broader assessment of phylogenetic relationships within the investigated clade.

Mountain spring ecosystems are recognised as important hotspots of freshwater biodiversity and frequently serve as refuges for diatom assemblages adapted to relatively stable environmental conditions. Low water temperatures with limited seasonal fluctuations and the presence of diverse microhabitats, including bryophyte-covered substrates, create conditions for the persistence of stenotopic taxa (Cantonati et al. 2012, 2022). Previous studies have demonstrated that mountain springs often support unique diatom floras and constitute important reservoirs of undescribed diversity (Cantonati and Lange-Bertalot 2010; Cantonati et al. 2023).

The integration of morphological and molecular analyses confirms *Odontidium fontinale* as a distinct species characteristic of spring habitats. Its documentation across two geographically isolated sites suggests a broader distribution rather than a strictly localised population. Morphologically, *O. fontinale* most closely resembles *O. mesodon*, particularly in valve outline and general valve architecture. Moreover, *O. mesodon* (syn. *Diatoma mesodon*) is frequently reported from spring ecosystems (Cantonati et al. 2006, 2012; Cantonati and Lange-Bertalot 2010; Falasco and Bona 2011; Battegazzore 2012; Żelazna-Wieczorek and Sulkowska 2024). The marked morphological similarity between these taxa raises the possibility that small specimens of *O. fontinale* may have previously been identified as *O. mesodon* in diatomological studies. Morphologically similar valves have previously been illustrated by Cantonati et al. (2022; fig. 3AC–AD, p. 14) and Noga et al. (2016; fig. 2f, p. 235). The specimen illustrated by Noga et al. (2016) was collected from the Terebowiec stream within the Bieszczady National Park, suggesting that morphologically similar forms may occur not only in spring habitats but also in flowing mountain stream waters. These records suggest that *O. fontinale* may have a broader ecological distribution than currently documented, although the identity of previously reported specimens should be verified before they can be attributed to this species. Likewise, the specimen illustrated by Kheiri et al. (2018; fig. 30, p. 365) and identified as *Tetracyclus rupestre* may represent a morphologically similar taxon.

The present study also highlights the importance of including environmental material in the description of new diatom taxa. The description should not be based only on cultured material, as cultivation conditions may affect valve dimensions and other morphological features. Valves of both *Odontidium fontinale* and *O. mesodon* from cultured material in this study were generally smaller than those observed in environmental samples. These differences indicate that cultured material may underestimate the morphological size range of a taxon and influence its differential diagnosis. However, both environmental and cultured material should be considered when assessing the morphological variability of newly described diatom species.

The incorporation of molecular evidence was therefore crucial for confirming the distinctiveness of the new species and demonstrates the value of integrative taxonomy in revealing overlooked diversity within freshwater araphid diatoms. The finding of *O. fontinale* sp. nov. suggests that species diversity within *Odontidium* may still be underestimated, particularly in poorly investigated spring ecosystems where morphologically similar taxa can remain overlooked without molecular analyses.

## Supplementary Material

XML Treatment for Odontidium fontinale

## Additional information

### Artificial Intelligence (AI) use

The authors accept full responsibility for the content of the manuscript, including the disclosure of any use of AI.

No AI tools were used in the preparation of this manuscript.

### Author ORCIDs

J. Sulkowska https://orcid.org/0009-0003-8456-1760

R.M. Olszyński https://orcid.org/0000-0003-0667-472X

C.E. Wetzel https://orcid.org/0000-0001-5330-0494

J. Żelazna-Wieczorek https://orcid.org/0000-0003-1044-8151

### Data availability

All of the data that support the findings of this study are available in the main text or Supplementary Information.

## Supplementary materials

Supplementary material 1 List of monoclonal strains included in the present study, with their taxonomic identification, sampling localities, and collection dates Data type docx https://binary.pensoft.net/file/1808690This dataset is made available under the Open Database License (http://opendatacommons.org/licenses/odbl/1.0/). The Open Database License (ODbL) is a license agreement intended to allow users to freely share, modify, and use this Dataset while maintaining this same freedom for others, provided that the original source and author(s) are credited. Julia Sulkowska, Rafał M. Olszyński, Carlos E. Wetzel, Joanna Żelazna-Wieczorek

Supplementary material 2 Measurements of selected traits of *Odontidium* spp. from environmental and cultured samples Data type xlsx https://binary.pensoft.net/file/1808691This dataset is made available under the Open Database License (http://opendatacommons.org/licenses/odbl/1.0/). The Open Database License (ODbL) is a license agreement intended to allow users to freely share, modify, and use this Dataset while maintaining this same freedom for others, provided that the original source and author(s) are credited.Author: Julia Sulkowska

Supplementary material 3 File containing the *rbc*L alignment Data type fasta https://binary.pensoft.net/file/1808692This dataset is made available under the Open Database License (http://opendatacommons.org/licenses/odbl/1.0/). The Open Database License (ODbL) is a license agreement intended to allow users to freely share, modify, and use this Dataset while maintaining this same freedom for others, provided that the original source and author(s) are credited.Author: Rafał M. Olszyński

Supplementary material 4 List of 83 diatom taxa used in the phylogenetic analysis, with GenBank accession numbers Data type xlsx https://binary.pensoft.net/file/1808693This dataset is made available under the Open Database License (http://opendatacommons.org/licenses/odbl/1.0/). The Open Database License (ODbL) is a license agreement intended to allow users to freely share, modify, and use this Dataset while maintaining this same freedom for others, provided that the original source and author(s) are credited.Author: Rafał M. Olszyński

Supplementary material 5 Morphometric and morphological characteristics of *Odontidium* species observed in cultured strains Data type docx https://binary.pensoft.net/file/1808694This dataset is made available under the Open Database License (http://opendatacommons.org/licenses/odbl/1.0/). The Open Database License (ODbL) is a license agreement intended to allow users to freely share, modify, and use this Dataset while maintaining this same freedom for others, provided that the original source and author(s) are credited. Julia Sulkowska, Rafał M. Olszyński, Carlos E. Wetzel, Joanna Żelazna-Wieczorek

Supplementary material 6 *Odontidium mesodon*LM culture Data type tif Explanation note Light microscope photomicrographs of *Odontidium mesodon* from culture. A–G'. Cleaned material. A–J. Strain ZBdPN_06; A–H. valve view; I–J. girdle view. K–W. Strain ZBdPN_32; K–W. valve view; W. girdle view. X–G'. Strain ZBdPN_34, valve view; X–W. valve view; W. girdle view. H'–K'. Living cells. L'–Q'. Strain ZBdPN_06; H'–K'. girdle view; L'–Q'. valve view. R'–B". Strains ZBdPN_32 and ZBdPN_34; R'–U'. girdle view; V'–B". valve view. Scale bar: 10 μm. https://binary.pensoft.net/file/1808695This dataset is made available under the Open Database License (http://opendatacommons.org/licenses/odbl/1.0/). The Open Database License (ODbL) is a license agreement intended to allow users to freely share, modify, and use this Dataset while maintaining this same freedom for others, provided that the original source and author(s) are credited. Julia Sulkowska, Rafał M. Olszyński

## References

[B1] Battegazzore M (2012) Environmental conditions of alpine springs of the upper Po River (NW Italy) on the basis of their epilithic diatom communities. Natural History Sciences 153(1): 49–62. 10.4081/nhs.2012.49

[B2] Beauger A, Wetzel CE, Voldoire O, Garreau A, Ector L (2017) Morphology and ecology of *Craticula lecohui* sp. nov. (Bacillariophyceae) from hydrothermal springs (Puy-de-Dôme, Massif Central, France) and comparison with similar *Craticula* species. Nova Hedwigia, Beiheft 146: 7–22. 10.1127/1438-9134/2017/007

[B3] Beauger A, Wetzel C, Allain E, Bertin C, Voldoire O, Breton V, Baker LA, Kolovi S, Biron D, Ector L (2022) *Chamaepinnularia salina* (Bacillariophyta), a new diatom species from French mineral springs (Massif Central). Phytotaxa 538(1): 55–73. 10.11646/phytotaxa.538.1.5

[B4] Bory de Saint-Vincent JBGM (1824) Diatome. *Diatoma*. In: Audouin I, Todd RB (Eds) Dictionnaire Classique d’Histoire Naturelle (Vol. 5). CRA-D., Rey et Gravier; Baudouin Frères, Paris, 461 pp. 10.5962/bhl.title.105726

[B5] Cantonati M, Lange-Bertalot H (2010) Diatom biodiversity of springs in the Berchtesgaden National Park (north-eastern Alps, Germany), with the ecological and morphological characterization of two species new to science. Diatom Research 25(2): 251–280. 10.1080/0269249X.2010.9705849

[B6] Cantonati M, Gerecke R, Bertuzzi E (2006) Springs of the Alps–sensitive ecosystems to environmental change: From biodiversity assessments to long-term studies. Hydrobiologia 562(1): 59–96. 10.1007/s10750-005-1806-9

[B7] Cantonati M, Angeli N, Bertuzzi E, Spitale D, Lange-Bertalot H (2012) Diatoms in springs of the Alps: Spring types, environmental determinants, and substratum. Freshwater Science 31(2): 499–524. 10.1899/11-065.1

[B8] Cantonati M, Angeli N, Spitale D, Lange-Bertalot H (2016) A new *Navicula* (Bacillariophyta) species from low-elevation carbonate springs affected by anthropogenic disturbance. Fottea 16(2): 255–265. 10.5507/fot.2016.013

[B9] Cantonati M, Bilous O, Spitale D, Angeli N, Segadelli S, Bernabè D, Lichtenwöhrer K, Gerecke R, Saber AA (2022) Diatoms from the spring ecosystems selected for the long-term monitoring of climate-change effects in the Berchtesgaden National Park (Germany). Water 14(3): e381. 10.3390/w14030381

[B10] Cantonati M, Casoria C, Gerecke R, Bilous OP, Maisto G, Segadelli S, Spitale D, Steinbauer A, Vogel S, Saber AA (2023) Diatom indicators of fluctuating/intermittent discharge from springs in two Bavarian nature conservation areas. Diversity 15(8): e915. 10.3390/d15080915

[B11] Dąbek P, Ashworth MP, Witkowski A, Li C, Bornman TG, Gonçalves V, Park J, Khim JS (2017) Towards a multigene phylogeny of the Cymatosiraceae (Bacillariophyta, Mediophyceae). I. Novel taxa within the subfamily Cymatosiroideae based on molecular and morphological data. Journal of Phycology 53(2): 342–360. 10.1111/jpy.12501 27988926

[B12] Falasco E, Bona F (2011) Diatom community biodiversity in an Alpine protected area: A study in the Maritime Alps Natural Park. Journal of limnology 70(2): 157–167. 10.4081/jlimnol.2011.157

[B13] Fray D, McGovern CA, Casamatta DA, Biddanda BA, Hamsher SE (2024) Metabarcoding reveals unique microbial mat communities and evidence of biogeographic influence in low‐oxygen, high‐sulfur sinkholes and springs. Ecology and Evolution 14(3): e11162. 10.1002/ece3.11162 PMC10961586 38529029

[B14] Jüttner I, Williams DM, Levkov Z, Falasco E, Battegazzore M, Cantonati M, Van de Vijver B, Angele C, Ector L (2015) Reinvestigation of the type material for *Odontidium hyemale* (Roth) Kützing and related species, with description of four new species in the genus *Odontidium* (Fragilariaceae, Bacillariophyta). Phytotaxa 234(1): 1–36. 10.11646/phytotaxa.234.1.1

[B15] Jüttner I, Williams DM, Gurung S, Van de Vijver B, Levkov Z, Sharma CM, Sharma SCM, Cox EJ (2017) The genus *Odontidium* (Bacillariophyta) in the Himalaya – a preliminary account of some taxa and their distribution. Phytotaxa 332(1): 1–25. 10.11646/phytotaxa.332.1.1

[B16] Kahlert M, Kelly MG, Mann DG, Rimet F, Sato S, Bouchez A, Keck F (2019) Connecting the morphological and molecular species concepts to facilitate species identification within the genus *Fragilaria* (Bacillariophyta). Journal of Phycology 55(4): 948–970. 10.1111/jpy.12886 31106407

[B17] Kheiri S, Solak CN, Edlund MB, Spaulding SA, Nejadsattari T, Asri Y, Hamdi SMM (2018) Biodiversity of diatoms in the Karaj River in the Central Alborz, Iran. Diatom Research 33(3): 355–380. 10.1080/0269249X.2018.1557747

[B18] Kociolek JP, Ashworth MP, Alverson AJ (2026) A phylogenetic classification of diatoms (Bacillariophyta). Journal of Phycology 62(1): 44–67. 10.1111/jpy.70125 41631365

[B19] Kützing FT (1844) Die Kieselschaligen Bacillarien oder Diatomeen. [I–VII], [1]-152 pp. [pls 1–30.] 10.5962/bhl.title.64360

[B20] Lai GG, Ector L, Lugliè A, Sechi N, Wetzel CE (2018) *Sellaphora gologonica* sp. nov. (Bacillariophyta, Sellaphoraceae), a new diatom species from a Mediterranean karst spring (Sardinia, Italy). Phytotaxa 356(2): 145–157. 10.11646/phytotaxa.356.2.4

[B21] Lai GG, Padedda BM, Wetzel CE (2025) *Mayamaea sardiniensis* sp. nov. (Bacillariophyceae), a new species from a thermomineral spring in Sardinia, Italy. Phytotaxa 696(2): 169–180. 10.11646/phytotaxa.696.2.6

[B22] Lange-Bertalot H, Hofmann G, Werum M, Cantonati M, Kelly MG (2017) Freshwater Benthic Diatoms of Central Europe: Over 800 Common Species Used in Ecological Assessment. Koeltz Botanical Books, Schmitten-Oberreifenberg, 942 pp.

[B23] Levkov Z, Williams DM (2011) Fifteen new diatom (Bacillariophyta) species from Lake Ohrid, Macedonia. Phytotaxa 30(1): 1–41. 10.11646/phytotaxa.30.1.1

[B24] Li CL, Ashworth MP, Witkowski A, Lobban CS, Zgłobicka I, Kurzydłowski KJ, Qin S (2016) Ultrastructural and molecular characterization of diversity among small araphid diatoms all lacking rimoportulae. I. Five new genera, eight new species. Journal of Phycology 52(6): 1018–1036. 10.1111/jpy.12460 27549783

[B25] Li Y, Nagumo T, Xu K (2022) Morphology and molecular phylogeny of *Amphora baotuensis* sp. nov., a new freshwater benthic diatom from a karst spring in China. Diatom Research 37(2): 145–153. 10.1080/0269249X.2022.2063390

[B26] Liu Y, Fu C, Wang Q, Stoermer EF (2010) A new species, *Diatoma rupestris*, from the Great Xing'An Mountains, China. Diatom research 25(2): 337–347. 10.1080/0269249X.2010.9705854

[B27] Medlin LK, Desdevises Y (2016) Phylogeny of “araphid” diatoms inferred from SSU and LSU rDNA, *rbc*L and *psb*A sequences. Vie et Milieu – Life and Environment 66: 179–200. https://hal.science/hal-02144540v1

[B28] Noga T, Stanek-Tarkowska J, Rybak M, Kochman-Kędziora N, Peszek Ł, Pajączek A (2016) Diversity of diatoms in the natural, mid-forest Terebowiec Stream, Bieszczady National Park. Journal of Ecological Engineering 17(4): 232–242. 10.12911/22998993/64506

[B29] Olszyński RM, Zakrzewski PK, Rimet F, Sulkowska J, Peszek Ł, Żelazna-Wieczorek J (2024) Morphology and phylogeny of *Nitzschia nandorii* sp. nov. (Bacillariophyceae), a new small–celled lanceolate species from a post-mining reservoir. PhytoKeys 241: 1–26. 10.3897/phytokeys.241.117406 PMC11009488 38618285

[B30] Olszyński RM, Mann DG, Zakrzewski PK, Peszek Ł, Ács É, Shemesh S, Trobajo R (2026) *Nitzschia excavata* sp. nov. (Bacillariaceae), a new diatom species from a post-mining reservoir revealed by morphology, molecular phylogeny, and metabarcoding-based biogeography. Scientific Reports 16(1): e16561. 10.1038/s41598-026-50312-9 PMC13219646 42209552

[B31] Rybak M, Peszek Ł, Górecka E, Zadrąg-Tęcza R, Levkov Z (2026) Morphological and molecular characterization of *Halamphora lukasiewiczii* sp. nov. (Bacillariophyceae, Amphipleuraceae), a new extremophilic diatom from petroleum seeps in the Polish Carpathians. PhytoKeys 271: 219–239. 10.3897/phytokeys.271.182323 PMC12997039 41858871

[B32] Sato S (2008) Phylogeny of araphid diatoms, inferred from morphological and molecular data. PhD Thesis, University of Bremen, Germany.

[B33] Theriot EC, Ashworth M, Ruck E, Nakov T, Jansen RK (2010) A preliminary multigene phylogeny of the diatoms (Bacillariophyta): Challenges for future research. Plant Ecology and Evolution 143(3): 278–296. 10.5091/plecevo.2010.418

[B34] Williams DM (1985) Morphology, taxonomy and inter-relationships of the ribbed araphid diatoms from the genera *Diatoma* and *Meridion* (Diatomaceae: Bacillariophyta). Bibliotheca Diatomologica 8: 1–228.

[B35] Williams DM (1990) Cladistic analysis of some freshwater araphid diatoms (Bacillariophyta) with particular reference to *Diatoma* and *Meridion*. Plant Systematics and Evolution 171(1): 89–97. 10.1007/bf00940597

[B36] Williams DM, Spaulding SA, Bishop I (2021) Studies on type material from Kützing’s diatom collection IV: The basionym, author and type of *Tetracyclus rupestre*. Phytotaxa 498(1): 44–50. 10.11646/phytotaxa.498.1.5

[B37] Winnicki T, Zemanek B (2001) Nature in the Bieszczady National Park. Bieszczady National Park, Ustrzyki Górne.

[B38] Żelazna-Wieczorek J (2011) Diatom flora in springs of Łódź Hills (Central Poland): Biodiversity, taxonomy, and temporal changes of epipsammic diatom assemblages in springs affected by human impact. Gantner Verlag, Ruggell.

[B39] Żelazna-Wieczorek J (2012) Okrzemki Bacillariophyta źródeł i odcinków źródłowych potoków w górnym odcinku rzeki San. Roczniki Bieszczadzkie 20: 220–229.

[B40] Żelazna-Wieczorek J, Knysak P (2017) Okrzemki (Bacillariophyta) źródła na Przełęczy Goprowskiej (Bieszczadzki Park Narodowy) w ocenie wpływu ruchu turystycznego. Roczniki Bieszczadzkie 25: 321–338.

[B41] Żelazna-Wieczorek J, Mostowik K (2019) Różnorodność gatunkowa okrzemek Bieszczadzkiego Parku Narodowego: okrzemki źródła Elizy na Połoninie Wetlińskiej. Roczniki Bieszczadzkie 27: 345–360.

[B42] Żelazna-Wieczorek J, Sulkowska J (2024) Ekosystemy źródlane na obszarach chronionych ostojami różnorodności gatunkowej okrzemek (Bacillariophyta). Roczniki Bieszczadzkie 32: 13–41.

