# Supplementary material for: Integrative taxonomy of a new Odontidium (Tabellariaceae) species from Poland: Combined morphological and molecular evidence
Source: PhytoKeys. 2026 Oct 14;280:51–72. doi: 10.3897/phytokeys.280.211531 (PMC13717476; doi:10.3897/phytokeys.280.211531)
Supplement: Supplementary file 1 [file phytokeys-280-051_article-211531__-s001.docx]

| Strain designation | ZBdPN_06 | ZBdPN_18 | ZBdPN_27 | ZBdPN_30 | ZBdPN_32 | ZBdPN_34 | ZBdPN_50 |
| --- | --- | --- | --- | --- | --- | --- | --- |
| Taxon | *Odontidium mesodon* (Ehrenberg) Kützing | *Meridion circulare* (Greville) C.Agardh | *Meridion circulare* (Greville) C.Agardh | *Odontidium* sp. | *Odontidium mesodon* (Ehrenberg) Kützing | *Odontidium mesodon* (Ehrenberg) Kützing | *Odontidium* sp. |
| Spring | Dwerniczek | Połonina Wetlińska | Goprowska Pass | Goprowska Pass | Goprowska Pass | Goprowska Pass | Tarnawa |
| Collection date | 06.09.2024 | 23.05.2025 | 22.05.2025 | 22.05.2025 | 22.05.2025 | 22.05.2025 | 09.09.2025 |
