# Supplementary material for: Integrative taxonomy of a new Odontidium (Tabellariaceae) species from Poland: Combined morphological and molecular evidence
Source: PhytoKeys. 2026 Oct 14;280:51–72. doi: 10.3897/phytokeys.280.211531 (PMC13717476; doi:10.3897/phytokeys.280.211531)
Supplement: Supplementary file 2 [file phytokeys-280-051_article-211531__-s005.docx]

| Taxa | *O. fontinalis* sp. nov. | *O. mesodon*  (Ehrenberg) Kützing | *O. minutum*  Levkov & D.M.Williams | *O. rostratum*  Levkov & Jüttner | *T. rupestre*  (Kützing) Grunow |
| --- | --- | --- | --- | --- | --- |
| Length [µm] | 9-16 | 10-40  18-33** | 12-16 | 10-58 | 4-28 |
| Width [µm] | 5-7 | 6-14  8-11** | 9-11 | 4.5-9 | 2-3*  3-12 |
| Striae [in 10 µm] | 20-28 | 22-25  21-26** | 24-30 | n.d. | 18-22 |
| Transapical ribs [in 10 µm] | 1-3 (4) | 3-6  3-5** | 2-5 | 3-4 | 3-5 |
| Rimoportula | 1 | 1 or 2 | 1 | 1 | 1 |
| Spines | Absent or 1 row | Present | Present | Present | Present |
| References | This study | Lange-Bertalot et al. 2017 | Levkov and Williams 2011 | Juttner et al. 2015 | Williams et al. 2021  Lange-Bertalot et al. 2017 |
